# A prospective study of monitoring practices for metabolic disease in antipsychotic-treated community psychiatric patients

**DOI:** 10.1186/1471-244X-7-28

**Published:** 2007-06-25

**Authors:** Paul Mackin, David R Bishop, Helen MO Watkinson

**Affiliations:** 1 School of Neurology, Neurobiology and Psychiatry Newcastle University Newcastle upon Tyne UK

## Abstract

**Background:**

Patients with severe mental illness are at increased risk for metabolic and cardiovascular disease. A number of recent guidelines and consensus statements recommend stringent monitoring of metabolic function in individuals receiving antipsychotic drugs.

**Methods:**

We conducted a prospective cohort study of 106 community-treated psychiatric patients from across the diagnostic spectrum from the Northeast of England to investigate changes in metabolic status and monitoring practices for metabolic and cardiovascular disease. We undertook detailed anthropometric and metabolic assessment at baseline and follow-up, and examined clinical notes and hospital laboratory records to ascertain monitoring practices.

**Results:**

A high prevalence of undiagnosed and untreated metabolic disease was present at baseline assessment. Mean follow-up time was 599.3 (SD ± 235.4) days. Body mass index (p < 0.005) and waist circumference (p < 0.05) had significantly increased at follow-up, as had the number of individuals who were either overweight or obese. Fifty-three per cent of individuals had hypertriglyceridemia, and 31% had hypercholesterolemia, but only 7% were receiving lipid-lowering therapy. Monitoring practices were poor. Recording of measures of adiposity occurred in 0% of individuals, and > 50% of subjects had neither blood glucose nor lipids monitored during the follow-up period.

**Conclusion:**

This cohort has a high prevalence of metabolic disease and heightened cardiovascular risk. Despite the publication of a number of recommendations regarding physical health screening in this population, monitoring rates are poor, and physical health worsened during the follow-up period.

## Background

Severe mental illness (SMI) is associated with a significant excess of physical co-morbidity and mortality [1,2], and as such represents a major public health concern. Previous studies have reported a high prevalence of undiagnosed and untreated metabolic disorders and cardiovascular risk factors in patients with SMI [3-7]. Consensus statements from the US [8] and UK [9] have recommended stringent monitoring of metabolic status and cardiovascular risk factors in psychiatric patients receiving antipsychotic drugs, and recently published UK guidelines from the National Institute for Health and Clinical Excellence (NICE) on the management of schizophrenia [10] and bipolar disorder [11] recognise the impact of physical co-morbidity in these mental disorders, as well as the paucity of high-quality research in this field. Currently available evidence suggests that effective screening and intervention for metabolic and cardiovascular disease is lacking [3,4,9,12].

The role of antipsychotic drugs, particularly the second generation (or atypical) agents, in the pathogenesis of metabolic dysfunction and CVD in SMI is controversial. There is a burgeoning literature examining this association, and current evidence suggests that some atypical agents (e.g. clozapine and olanzapine) may have a more deleterious profile with regard to metabolic dysfunction than others (e.g. quetiapine and risperidone) [8,9,13]. Notwithstanding the reported association between antipsychotic drugs and metabolic dysfunction, prospective, longitudinal studies investigating the evolution of metabolic disease in this population are sparse. A number of recent guidelines recommend switching an antipsychotic drug to an agent with a less deleterious effect on metabolic function in patients who develop hyperglycaemia or experience significant weigh gain [8,9,14], but in the absence of longitudinal data, such strategies lack a firm evidence base.

We prospectively studied metabolic function in a cohort of antipsychotic-treated community psychiatric patients from across the diagnostic spectrum, and investigated rates of monitoring and intervention for metabolic and cardiovascular risk.

## Methods

We recruited 106 patients from psychiatric out-patient clinics in the Northeast of England between January 2002 and March 2004. Exclusion criteria and baseline characteristics of this cohort have previously been described [4]. Briefly, the only entry criteria were that the patient was prescribed an antipsychotic drug (typical, atypical or combination) and was clinically stable. Patients who had anorexia nervosa or bulimia, those who were actively misusing substances or alcohol, and those with known malignant disease were excluded. All the secondary care consultant psychiatrists and primary care physicians responsible for the mental and physical healthcare of these patients were sent a detailed summary of the metabolic parameters obtained following the baseline visit, and any abnormal findings were highlighted. All patients were invited to participate in this prospective study between June 2005 and December 2005 as part of a planned 18-month follow-up assessment. Subjects gave written informed consent to participate in this study which was approved by the Newcastle Local Research Ethics Committee.

Participants were given written instructions to fast overnight on the day before assessment, and subjects were asked to confirm their fasting status by a member of the research team on the morning of study. All assessments were performed between 8.30 am and 10.00 am on the study day. Demographic and illness characteristics were recorded, together with family history of type 2 diabetes and cardiovascular disease. Current medication (including non-psychotropic drugs) and dosage was recorded and confirmed, where necessary, by reference to case notes and general practitioner records.

Height, weight, and waist and hip circumference were recorded using standardised anatomical landmarks. A single venous blood sample was withdrawn and analysed for glucose, HbA_1c_, insulin and lipid profile (total cholesterol, high-density lipoprotein (HDL-C) and low-density lipoprotein (LDL-C) cholesterol and triglycerides). Insulin was measured by ELISA. The homeostatic model assessment [15] was used to assess glucose handling and values calculated using the HOMA calculator, version 2.2 (^© ^Diabetes Trial Unit, University of Oxford). Impaired fasting glucose (IFG) was defined as fasting blood glucose between 6.1 and 7.0 mmol/l, and diabetes mellitus as fasting blood glucose > 7.0 mmol/l [16].

All patients' notes were screened for evidence of monitoring of metabolic function and recommendations regarding lifestyle and referrals to other healthcare professionals during the follow-up period. Hospital biochemistry laboratory records were checked to confirm whether blood glucose or lipid analyses had been requested by the psychiatrist or a primary care physician, but had not been recorded in the case-notes.

Data analysis was conducted using the Statistical Package for the Social Sciences, version 11, [17]. Comparisons between baseline and follow-up characteristics were examined by t-test, chi-squared or McNemar tests where appropriate. All reported p values are two-tailed. Statistical significance is defined as p < 0.05.

## Results

### Patient characteristics

Of the original 106 patients in the baseline cohort, 90 (85%) consented to participate in the current study. Six (5.7%) did not reply to the invitation, 6 (5.7%) refused to consent, 2 (1.9%) were too unwell to participate, and 1 patient (1%) denied having participated in the original study. Baseline and follow-up characteristics of the 90 patients are given in table 1. Forty-four (49%) of subjects were male and 46 (51%) were female. Eighty-eight (98%) were Caucasian. With regard to diagnosis, 32 (35.6%) had bipolar disorder, 27 (30.0%) had schizophrenia, 9 (10.0%) had schizo-affective disorder, and 22 (24.4%) had other mood and anxiety disorders. Mean duration between the baseline and follow-up visits was 599.3 days (SD ± 235.4; Range 328–1175). Of note is the high prevalence of family history of both cardiovascular disease and diabetes mellitus in the first-degree relatives of subjects.

**Table 1:** Characteristics of 90 patients at baseline and follow-up assessment.

	Baseline	Follow-up
	(n = 90)	(n = 90)

**Age, **years	44.2 (11.7)	45.8 (11.8)
**Duration of illness, **months	212.6 (154.8)	230.6 (161.2)
**Smoking, **%	40	40
**Cigarettes **n/day	12.1 (15.5)	10.7 (15.9)
**Alcohol **units/day	5.6 (9.2)	6.8 (12.3)
**Current substance misuse **(%)	32.2	30
**Family history of DM **(%)	30.0	34.4
**Family history of CVD **(%)	58.9	61.0

DM – Diabetes mellitus; CVD – cardiovascular disease

Data are means ± SD unless otherwise stated

### Medication

Of the 90 patients prescribed antipsychotic medication at baseline, 83 (92%) were still taking an antipsychotic drug at follow-up. Sixty-eight (82%) patients were prescribed the same antipsychotic regimen as at baseline assessment. Details of individual drugs are given in table 2.

**Table 2:** Antipsychotic, and non-antipsychotic medication taken by patients with SMI

Drug	n (%)
**Antipsychotic **(n = 90)	
Yes	83 (92.2)
No	7 (7.8)
One antipsychotic	71 (78.8)
Combination antipsychotic	12 (13.3)
	
**Typical or atypical agent **(n = 71)	
**Typical**	**16 (22.5)**
Zuclopenthixol	2 (2.8)
Flupenthixol	5 (7.0)
Haloperidol	1 (1.4)
Fluphenazine	1 (1.4)
Pipothiazine	1 (1.4)
Sulpiride	4 (5.6)
Trifluoperazine	2 (2.8)
**Atypical**	**55 (77.5)**
Amisulpiride	3 (4.2)
Clozapine	7 (9.9)
Olanzapine	29 (40.8)
Quetiapine	8 (11.3)
Risperidone	8 (11.3)
	
**Non-antipsychotic psychotropic drugs **(n = 90)	
**Antidepressants**	
SSRI	29 (32.2)
SNRI	9 (10)
NaSSA	4 (4.4)
TCA	7 (7.8)
MAOI	3 (3.3)
**Mood Stabiliser**	
Valproate	16 (17.8)
Lamotrigine	8 (8.9)
Carbamazepine	1 (1.1)
Lithium	15 (16.7)
**Other**	
Gabapentin	2 (2.2)
Benzodiazepines	28 (31.1)
Tryptophan	2 (2.2)
Anticholinergic agent	15 (16.7)
	
**Non-psychotropic drugs **(n = 90)	
Antihypertensive agent	13 (14.4)
Thyroxine	10 (11.1)
Hypoglycaemic agent	4 (4.4)

### Metabolic parameters

Comparisons between baseline and follow-up metabolic parameters are given in table 3. Both BMI and waist circumference were significantly increased at follow-up, and a greater proportion of subjects were classified as overweight (BMI ≥ 25 kg/m^2^) compared with follow-up. In the whole cohort, pancreatic β-cell function and insulin resistance estimated by HOMA were significantly lower at follow-up compared with baseline, but these parameters were not significantly different when those subjects no longer prescribed antipsychotic drugs (n = 7) were removed from the analysis. A high proportion of patients had dyslipidaemias at baseline (hypercholesterolemia = 27.8%; hypertriglyceridemia = 53.3%), and continued to do so at follow-up (hypercholesterolemia = 28.9%; hypertriglyceridemia = 51.1%). At follow-up, 30 patients (33.3%) met criteria for the metabolic syndrome as defined by the International Diabetes Federation [18]. A comparison between rates of metabolic syndrome at baseline and follow-up was not possible as blood pressure readings were not available from the baseline assessment.

**Table 3:** Comparison of baseline and follow-up metabolic parameters.

	Baseline	Follow-up	Follow-up
	(n = 90)	(all subjects, n = 90)	(antipsychotic-treated subjects, n = 83)

**BMI**, kg/m^2^	29.2 (5.1)	29.9 (4.9)*	29.8 (4.9)*
**BMI category **			
Underweight (%)	1.1	2.2	2.4
Normal (%)	16.7	10.0	8.4
Overweight (%)	34.4	43.3*	44.6*
Obese (%)	47.8	44.4	44.6
**Waist Circumference**, cm	94.6 (13.2)	96.2 (13.1) †	96.2 (13.0) †
**Fasting blood glucose**, mmol/l	5.6 (2.4)	5.5 (1.4)	5.5 (1.4)
**HbA**_1c_, %	5.4 (1.2)	5.6 (1.0)	5.6 (1.0)
**Serum insulin**, MU/l	12.9 (13.4)	11.1 (8.1)	11.1 (8.2)
**HOMA B**, %	106.3 (38.6)	98.8 (38.8) †	99.7 (39.7)
**HOMA S**, %	95.9 (60.5)	98.6 (55.6)	98.4 (55.6)
**HOMA IR**, %	1.8 (1.7)	1.5 (1.1) †	1.5 (1.1)
**Glycaemic status**			
Normoglycaemia, %	86.7	85.6	86.8
Impaired fasting glucose, %	6.7	8.9	7.2
Diabetes Mellitus, %	6.7	5.6	6.0
**Total Cholesterol**, mmol/l	5.6 (1.2)	5.7 (1.5)	5.7 (1.5)
**Raised total cholesterol**, %	27.8	28.9	31.3
**HDL-Cholesterol**, mmol/l	1.3 (0.4)	1.3 (0.4)	1.3 (0.4)
**LDL-Cholesterol**, mmol/l	3.2 (0.9)	3.3 (1.1)	3.3 (1.3)
**Triglycerides**, mean (range), mmol/l	2.3 (0.6–8.4)	2.1 (0.5–6.9)	2.1 (0.5–6.9)
**Raised total triglycerides**, %	53.3	51.1	53.0
**Lipid-lowering therapy**, %	4.4	7.8	7.2

* p < 0.005 baseline vs follow-up; † p < 0.05 baseline vs follow-up

Data are means ± SD unless otherwise stated.

Prescribing rates of lipid-lowering therapies were low (4.4% and 7.8% at baseline and follow-up, respectively).

### Monitoring

Details of records in the case notes of BMI, identified weight problems and referrals to other health care professionals for lifestyle or medical intervention are given in table 4, along with the proportion of subjects who received blood glucose and/or lipid monitoring during the follow-up period. Monitoring of metabolic status was poor across all domains, the majority of patients receiving no assessment of metabolic function (BMI, waist circumference, blood glucose or lipid measurement) during the follow-up period.

**Table 4:** Monitoring of metabolic parameters in 90 patients over an 18-month follow-up period.

	Number of patients monitored
	(%)
**BMI**	
Yes	0
No	100
**Waist circumference**	
Yes	0
No	100
**Weight problem identified**	
Yes	21.6
No	78.4
**Lifestyle advice offered**	
Yes	9.5
No	90.5
**Referral to health care professional **	
Practice nurse	4.0
Dietician	1.3
Other physician	1.3
None	93.3
**Blood monitoring**	
Glucose and lipids	26.7
Glucose only	13.3
Lipids only	8.9
Neither	51.1
**> 12 months since monitoring**	
Glucose	67.8
Lipids	72.2

## Discussion

At baseline there was a high prevalence of overweight, obesity and dyslipidaemias in this population; a significant proportion of patients also had undiagnosed disorders of glucose homeostasis, and treatment rates for these metabolic disorders was low [4]. Despite informing individual primary and secondary care physicians of these baseline results, after a mean follow-up period of 19.2 months, the metabolic parameters of this cohort had either worsened or remained unchanged.

Mean BMI had significantly increased during the follow-up period, as had the number of individuals who were in the overweight or obese categories. Waist circumference, a measure of visceral adiposity closely associated with Type 2 diabetes [19] and cardiovascular risk [20], had also significantly increased compared with baseline values. A high proportion of patients had elevated total cholesterol and/or triglycerides at baseline assessment, and a similar proportion had dyslipidaemias at follow-up assessment. More than 50% of individuals had raised triglycerides, and almost 30% had elevated total cholesterol at follow-up, but only 7% were prescribed lipid-lowering therapy, which was marginally increased from 4% at baseline. Taken together, these data suggest that the metabolic status of this cohort of patients, all of whom are in contact with mental health services, is worsening over time, and appropriate intervention appears to be lacking. Given the established increased risk of cardiovascular morbidity and mortality in individuals with severe mental illness [21], this is a worrying and ominous trend.

The metabolic health of these patients and poor rates of intervention may be explained, at least in part, by inadequate monitoring practices. A number of recent guidelines and consensus documents have highlighted the need for close monitoring of metabolic function in this population in order to minimise cardiovascular risk [9-11]. Regular monitoring of measures of adiposity and serum glucose and lipid estimation is recommended by all these documents.

Our data reveal an alarmingly poor rate of monitoring of metabolic parameters; BMI or waist circumference was not recorded in the psychiatric case notes of any patient. Although the presence of a weight-problem was identified in 21% of patients there was documented evidence of lifestyle advice in less than 10%, and only 7% were referred to another healthcare professional for further intervention. Monitoring of blood glucose and lipid levels was also poor. The majority of patients (51%) had not received blood glucose or lipid monitoring during the follow-up period; only 27% of individuals had received both glucose and lipid monitoring. Where glucose and lipid monitoring had been performed, for the majority of patients this had not taken place within the preceding 12 months. Although we were able to establish the proportion of patients who had undergone blood monitoring during the follow-up period from hospital laboratory databases, the vast majority of these results (> 90%) were not recorded in the psychiatric case notes indicating that psychiatrists were unaware of the metabolic status of these individuals.

Another observation of interest is the high reported prevalence of a positive family history of both type 2 diabetes (34%) and cardiovascular disease (61%) in the first degree relatives of subjects. The high prevalence of type 2 diabetes (18–30%) in mentally well first-degree relatives of individuals with schizophrenia has previously been reported [22], leading to speculation that there may be common susceptibility regions within the genome for both schizophrenia and type 2 diabetes [23]. Shared exposure to environmental influences known to be involved in the pathogenesis of diabetes may also partly explain this relationship. We are not aware of any previous data reporting a high prevalence of cardiovascular disease in first degree relatives of patients with severe mental illness. The prevalence of type 2 diabetes in relatives together with other shared genetic and environmental influences may explain this association, but this is an area of investigation which warrants further research.

This study has a number of limitations. Subjects were recruited from secondary and tertiary care mental health outpatient clinics, and all volunteered to participate. This cohort is, therefore, likely to comprise a group of relatively well motivated individuals who may take a more active interest in their physical (and mental) well-being than those individuals who were not in contact with mental health services or who refused to participate in this study. The physical health of this cohort may, therefore, not be representative of all psychiatric out-patients. In fact, it is probable that the physical health of those individuals who are not in contact with mental health services or who refuse to participate in physical health screening studies is less closely monitored, and therefore this population will have an even greater burden of physical co-morbidity and cardiovascular risk. The fact that subjects were recruited from a single well-defined geographical region within the UK may raise questions about extrapolating these data to other populations. However, primary and secondary care health service configurations within this geographical region are typical for the UK, and subjects were recruited from a number of community mental health centres representing a diverse socioeconomic population. A further limitation of this study is the lack of data on specific drug-effects on metabolic function. This study was not designed, nor indeed powered, to investigate the effects of individual antipsychotic drugs, or classes of drugs, on the evolution of metabolic dysfunction; our aim was to investigate monitoring practices for metabolic and cardiovascular risk and to describe the change over time of metabolic function in a diagnostically diverse cohort of patients treated with any antipsychotic drug.

## Conclusion

In summary, we have presented data from a prospective study on monitoring practices for metabolic function in a cohort of antipsychotic-treated outpatients with severe mental illness from across the diagnostic spectrum. At baseline assessment there was a high prevalence of overweight, obesity and dyslipidaemias. Despite notifying relevant healthcare professionals about the extent of physical morbidity in this population, and the existence of a number of recently published guidelines and recommendations on physical health monitoring in patients receiving antipsychotic drugs, metabolic parameters were either unchanged or, in some cases, significantly worse, at follow-up assessment. Rates of monitoring of measures of adiposity, blood glucose and lipid levels, referrals to other healthcare professionals for medical or lifestyle intervention, and treatment for metabolic diseases is poor, and in many cases non-existent. There is a clear and urgent need to develop integrated models of care which specifically target the physical health of this population which already suffers an excess burden of physical morbidity and mortality.

## Abbreviations

SMI Severe mental illness

NICE National Institute for health and Clinical Excellence

CVD Cardiovascular disease

HbA_1c _Glycosylated haemoglobin

HDL-C High-density lipoprotein cholesterol

LDL-C Low-density lipoprotein cholesterol

ELISA Enzyme-linked immunosorbent assay

HOMA Homeostatic Model Assessment

IFG Impaired fasting glucose

## Competing interests

PM has received honoraria for educational meetings from AstraZeneca, BristolMyers Squibb, Eli Lilly and Jannsen-UK. The other authors have no competing interests.

## Authors' contributions

PM designed the protocol, assisted in data collection, analysed the data and prepared this manuscript. DRB assisted in data collection, analysis of the results and preparation of the manuscript. HMOW assisted in developing the experimental protocol, data collection and preparation of the manuscript. All authors have seen and approved the final version of the manuscript.

## Pre-publication history

The pre-publication history for this paper can be accessed here:

http://www.biomedcentral.com/1471-244X/7/28/prepub
